# Rapid Differential Detection of Japanese Encephalitis Virus and Getah Virus in Pigs or Mosquitos by a Duplex TaqMan Real-Time RT-PCR Assay

**DOI:** 10.3389/fvets.2022.839443

**Published:** 2022-04-07

**Authors:** Yan Zhang, Yuhao Li, Zhixin Guan, Yang Yang, Junjie Zhang, Qing Sun, Beibei Li, Yafeng Qiu, Ke Liu, Donghua Shao, Zhiyong Ma, Jianchao Wei, Peng Li

**Affiliations:** ^1^College of Animal Science, Yangtze University, Jingzhou, China; ^2^Shanghai Veterinary Research Institute, Chinese Academy of Agricultural Sciences, Shanghai, China

**Keywords:** Getah virus, Japanese encephalitis virus, duplex TaqMan real-time RT-PCR, differentiation, mosquitos, pigs

## Abstract

Both JEV (Japanese encephalitis virus) and GETV (Getah virus) pose huge threats to the safety of animals and public health. Pigs and mosquitoes play a primary role in JEV and GETV transmission. However, there is no way to quickly distinguish between JEV and GETV. In this study, we established a one-step duplex TaqMan RT-qPCR for rapid identification and detection of JEV and GETV. Primers and probes located in the *NS1* gene of JEV and the *E2* gene of GETV that could specifically distinguish JEV from GETV were selected for duplex TaqMan RT-qPCR. In duplex real-time RT-qPCR detection, the correlation coefficients (R^2^) of the two viruses were higher than 0.999. The RT-qPCR assay demonstrated high sensitivity, extreme specificity, and excellent repeatability. Detection of JEV and GETV in field mosquito and pig samples was 100 times and 10 times more sensitive than using traditional PCR, respectively. In addition, the new test took less time and could be completed in under an hour. Clinical sample testing revealed the prevalence of JEV and GETV in mosquitoes and pig herds in China. This complete duplex TaqMan RT-qPCR assay provided a fast, efficient, specific, and sensitive tool for the detection and differentiation of JEV and GETV.

## Introduction

Getah virus (GETV) is a mosquito-borne, enveloped, single-stranded, positive-sense RNA virus belonging to the genus *Alphavirus* in the family *Togaviridae* that was first isolated in Malaysia in 1955 from *Culex* spp. mosquitoes (1). Subsequent studies indicated that this virus has a broad geographic distribution in Southeast Asia and Eurasia, including China, Thailand, Japan, and Russia (2). GETV mainly infects horses and pigs, which play a critical role in the amplification and circulation of the virus; the virus is also pathogenic to foxes and cattle and can cause fever in humans (3). Horses infected with GETV can suffer fever, swollen hind limbs, skin rashes, and other symptoms (4). To date, there have been five horse-based GETV outbreaks in Japan, in 1978, 1979, 1983, 2014, and 2015, resulting in large economic losses (5). GETV infection can cause fetal death and reproductive disorders in pigs. Approximately 70 GETV isolates have been identified in China, which are widespread in 15 provinces including Hunan, Shanghai, Sichuan, Henan, and Shandong (6). In 2017, GETV broke out in a pig farm in Hunan, causing serious economic losses (3). At the same time, the positive detection rate of GETV antibodies in most areas of China continues to rise, and GETV has even been found in commercial live vaccines and boar semen, which poses a potential threat to animal husbandry and public health (7).

Japanese encephalitis virus (JEV) is a mosquito-borne virus that belongs to the genus *Flavivirus* in the family *Flaviviridae* and is distributed throughout East, Southeast, and South Asia (8). JEV causes public health problems as a major etiological agent of viral encephalitis, with data revealing more than 67,900 cases of Japanese encephalitis caused by JEV each year throughout the world and a mortality rate as high as 20% to 30% (9). JEV is transmitted between birds and swine in enzootic cycles with mosquito vectors (mainly *Culex* spp. mosquitoes) (10). Pigs are considered the most important natural amplification host (11). JEV infection in pigs causes reproductive disorders in pregnant sows such as miscarriage and stillbirth, and piglets will show obvious neurological symptoms (12). Except for Qinghai and Tibet, all other provinces in China have reported JEV incidence (13).

JEV and GETV cause similar clinical symptoms and pathological changes in pigs and spread through the same medium. In recent years, they have caused huge economic losses worldwide. Tajima et al. (14) detected both JEV and GETV in pig serum, confirming that these two viruses are indeed circulating in pigs. However, due to a lack of therapeutics, early detection and treatment are key to the prevention and control of JEV and GETV (15, 16). Therefore, making a rapid and effective distinction between JEV and GETV is essential for the early prevention and treatment of these two viruses.

Virus isolation tests are the gold standard for virus testing. However, this approach is laborious and time-consuming (17). MAC-ELISA is another traditional method for detecting viruses, although this method is more sensitive than virus isolation, there may be cross-reactions between viruses, especially flaviviruses, which reduces the accuracy of this method. RT-qPCR has gradually become a popular method for virus detection due to its rapidity, sensitivity, and accuracy (18). TaqMan RT-qPCR and SYBR Green-based RT-qPCR have been developed separately to detect JEV and GETV (15, 19, 20). However, few studies have used the TaqMan RT-qPCR method to simultaneously quantitatively detect and distinguish JEV and GETV strains from field specimens.

Therefore, it is necessary to establish a method that can quickly distinguish and detect JEV and GETV in pigs or mosquitoes. This is of great significance for the rapid detection, virological monitoring, clinical diagnosis, epidemiological investigation, and decision-making process of these two viruses.

## Materials and Methods

### Primer and Probe Design

To design efficient primer pairs and probes, conserved regions of the viral genome were identified in JEV and GETV strains. The nucleotide sequence of the *NS1* gene of JEV reference strain N28 was obtained from GenBank (no. MH753126) and compared with the *NS1* gene sequence of other JEV strains (JN381843, HM366552, KT957423, KU508408, KC183732, JN711458, AF075723, KX945367, MN544779). The nucleotide sequence of the *E2* gene of GETV reference strain M1 was obtained from GenBank (no. EU015061) and compared with the *E2* gene sequence of other GETV strains (EU015063, KY434327, KY450683, EU015062, MG869691, EF631998). Nucleotide sequence data were retrieved from the GenBank database and CLUSTAL W algorithm implemented in the MegAlign of DNASTAR program package (MegAlign 5.00, DNASTAR Inc., Madison, USA).Oligonucleotide primers and probes used for JEV and GETV real-time amplification were designed using SnapGene software (SnapGene^®^4.1.9) software against the non-structural gene (*NS1*) of JEV and the structural gene (*E2*) of GETV. Potential target regions were selected, and primers were synthesized and evaluated for use in quantitative real-time RT-PCR. The nucleotide sequences of the JEV- and GETV-specific primer pairs and probes, and the characteristics of the amplicons are shown in Table 1.

**Table 1:** Oligonucleotide primers and fluorogenic probes used in the duplex TaqMan RT-qPCR assay.

**Virus**	**Location**	**Primer/probe**	**Sequences**	**Length (bp)**
JEV	NS1	Primer-JEV-F	GGGCCTTCTGGTGATGTTT	80
		Primer-JEV-R	AAACCGCAGGAATVGTCAAT	
		Probe-JEV	^FAM−^TCGCAAGAGGTGGACGGCCA^−*BHQ*−1^	
GETV	E2	Primer-GETV-F	AAGTGGCAGTACACCTCCTC	92
		Primer-GETV-R	GTGGAGTTGGTCAGAGGGAA	
		Probe-GETV	^HEX−^AGAGCCGACCAGTTGTCTCGCA^−*BHQ*−1^	

*Numbering based on MH753126 (N28, JEV) and EU015061 (M1, GETV). V = A/G/C*.

### Standard Strains and Clinical Samples

The JEV reference strain N28 (GenBank no. MH753126) and GETV reference strain M1 (GenBank no. EU015061) were procured from the Shanghai Veterinary Research Institute, Chinese Academy of Agricultural Sciences (17). The JEV strain was grown and titered in newborn hamster kidney cells (BHK-21), which were maintained in Dulbecco's modified Eagle's medium (DMEM; Gibco, Grand Island, NY, USA) containing 10% fetal bovine serum (FBS) (Gibco) at 37°C in an atmosphere containing 5% CO_2_. The GETV strain was grown and titered in *Aedes albopictus* C6/36 cells, which were maintained in RPMI 1640 (Gibco) containing 10% FBS (Gibco) at 28°C in an atmosphere containing 5% CO_2_.

Classical swine fever virus (CSFV, KT119352), porcine parvovirus (PPV, OG155649), pseudorabies virus (PRV, AF218843), and porcine reproductive and respiratory syndrome virus (PRRSV, JN662424) were provided by Shanghai Veterinary Research Institute, Chinese Academy of Agricultural Sciences. The genome sequence of African swine fever virus (ASFV, CM033491) was provided by the National Research Center for Exotic Animal Diseases, China Animal Health and Epidemiology Center.

During June, July and August 2016, a total of 15 pig samples (blood and tissue) and 1834 mosquitoes were collected from Shanghai pig farms and horse farms. Adult mosquitoes were collected by placing mosquito traps (Gongfu Xiaoshuai, Wuhan, China) around the pigs and stables from 6:00 pm to 6:00 am. Live mosquitoes were killed by freezing. Female mosquitoes were sorted and pooled by species into groups of 100 mosquitoes per pool and kept at −80°C for further use. These samples were used to compare the performance of duplex TaqMan RT-qPCR with conventional RT-PCR and virus isolation.

During 2017–2018, 331 samples (including pig brain, blood and mosquitoes) were collected from pig farms in Xinjiang, Yunnan and Shanghai, China. Pig tissue samples were provided by the China Animal Health and Epidemiology Center. Mosquito samples were collected from 12 different pig farms by the JEV research team at Shanghai Veterinary Research Institute. Animal welfare and experimental procedures were carried out in accordance with the Guide for the Care and Use of Laboratory Animals, and animal ethics approval was obtained from the Committee of Shanghai Veterinary Research Institute, Chinese Academy of Agricultural Sciences, Shanghai.

All mosquito pools were placed in 2 mL tubes; 1 mL DMEM was added, and samples were then homogenized, freeze-thawed, and centrifuged at 12,000 rpm for 20 min at 4°C. Swine samples were homogenized with DMEM medium. This 10% (w/v) suspension was centrifuged at 5,000 rpm for 10 min at 4°C to obtain the supernatant. Supernatants were collected for further RNA extraction and JEV isolation (17).

### RNA Extraction and Reverse Transcription

All clinical samples were resuspended in 0.9% sodium chloride solution (Shanghai Xin Yu Biotech Co., Ltd). RNA was extracted from 200 μL homogenized sample using Magnetic bead method nucleic acid extraction kit (Hangzhou Bioer Technology Co., Ltd) following the manufacturer's instructions. An Evo M-MLV Reverse Transcription Kit (Accurate Biotechnology Co., Ltd.) was used to reverse transcribe the extracted RNA into cDNA according to the manufacturer's instructions. cDNA products were stored at −20°C until further study.

### Construction of the Two-Step Dual TaqMan RT-qPCR Detection Method

The real-time RT-PCR assays in this study were performed using a Premix Ex Taq^TM^(Probe qPCR) kit (TaKaRa, Dalian, China). According to the instructions of Premix Ex Taq^TM^(Probe qPCR) kit, we set the primer concentration to 0.2 μM (0.4 μL). Then we explored the probe concentrations for JEV and GETV in the duplex RT-qPCR method (0.15 μM/0.2 μM/0.25 μM). The final results showed that the method was most sensitive to the two positive samples when the probe concentrations for JEV and GETV were 0.15 and 0.25 μM (0.3 and 0.5 μL), respectively (Supplementary Figure 1). The total reaction volume was 20 μL with 2 μL template cDNA, the same amount of primer sets and probes for detecting JEV and GETV (each primer 0.4 μL, probe 0.3 μL/0.5 μL), 10 μL 2 × TaqMan Mix, RNase-free water made up to 20 μL. Duplex RT-qPCR based on TaqMan amplification was performed using a CFX96 Real-Time System (Bio-Rad) with the following amplification conditions: 95°C for 1 min, followed by 35 cycles of 95°C for 15 s and 60°C for 30 s. The primer sets and specific probes were tested in a single-plex real-time qPCR reaction, and then in a double qPCR reaction.

### Construction of the One-Step Dual TaqMan RT-qPCR Detection Method

Based on the established two-step duplex RT-qPCR prevention, this study further established a one-step duplex TaqMan RT-qPCR using the One Step PrimeScript™ RT-PCR kit (TaKaRa, Dalian, China). The total reaction volume was 20 μL with 2 μL template RNA, the same amount of primer sets and probes for detecting JEV and GETV (each primer 0.4 μL, probe 0.3 μL and 0.5 μL), 10 μL 2 × One-Step Probe StarScript II Buffer, 2 μL StarScript II One-Step Probe Enzyme Mix, RNase-free water made up to 20 μL. One-step RT-qPCR based on TaqMan amplification was performed using a CFX96 Real-Time System (Bio-Rad) with the following amplification conditions:50°C for 5 min, 95°C for 1 min, followed by 35 cycles of 95°C for 15 s and 60°C for 15 s. Optimal concentrations of primer sets and specific probes were explored in a two-step RT-qPCR reaction and tested in a one-step RT-qPCR.

### Standard Plasmid Construction and Standard Curve Generation

The *NS1* gene of JEV and the *E2* gene of GETV were inserted into the pMD-19-T vector and transcribed into RNA using a mMESSAGE mMACHINE® T7 Kit (Thermo Fisher Scientific). RNA was quantified using a NanoDrop 1000 (Thermo Fisher Scientific), and the copy number of the recombinant plasmid was calculated using the following formula: copies/μL = 6 × 10^23^× ssRNA (ng/μL) × 10^−9^÷molecular weight (g/mol) (21). Serial 10-fold dilutions of RNA standard products (10^9^ copies/μL~10^1^ copies/μL) were prepared and stored at −80°C until use. At the same time, the RNA standard was converted into cDNA to evaluate and compare the sensitivity of RT-qPCR and conventional RT-PCR detection. Using the prepared 10-fold serial dilution standards as template, a double qPCR method was developed to establish a standard curve for detecting JEV and GETV.

### Specificity and Sensitivity Analysis of the Duplex TaqMan RT-qPCR Assay

To determine the specificity of the method, the following viruses were used in specific experiments: JEV, GETV, CSFV, PPV, PRV, PPRRSV, and ASFV. For sensitivity assays, the JEV clone plasmid was subjected to 10-fold gradient dilution for TaqMan real-time RT-PCR, and results were compared with those of the traditional RT-PCR method and singleplex TaqMan-based real-time PCR.

### Repeatability Detection of Duplex TaqMan RT-qPCR Assay

The newly established method was used to test the intra-assay (each sample was tested three times in a reaction) and inter-assay (the same sample was subjected to RT-qPCR reaction every other month, a total of three times) repeatability. Ct values and coefficients of variation (CV, %) were analyzed to evaluate the repeatability of the method.

### Testing of Clinical Samples

The real-time RT-qPCR assay developed in this study was used to detect JEV and GETV in clinical samples to verify the feasibility of the method for clinical application and to confirm that the method has the ability to differentiate and detect JEV and GETV.

## Results

### Design of Specific Primers and Probes

**Figure 1:**
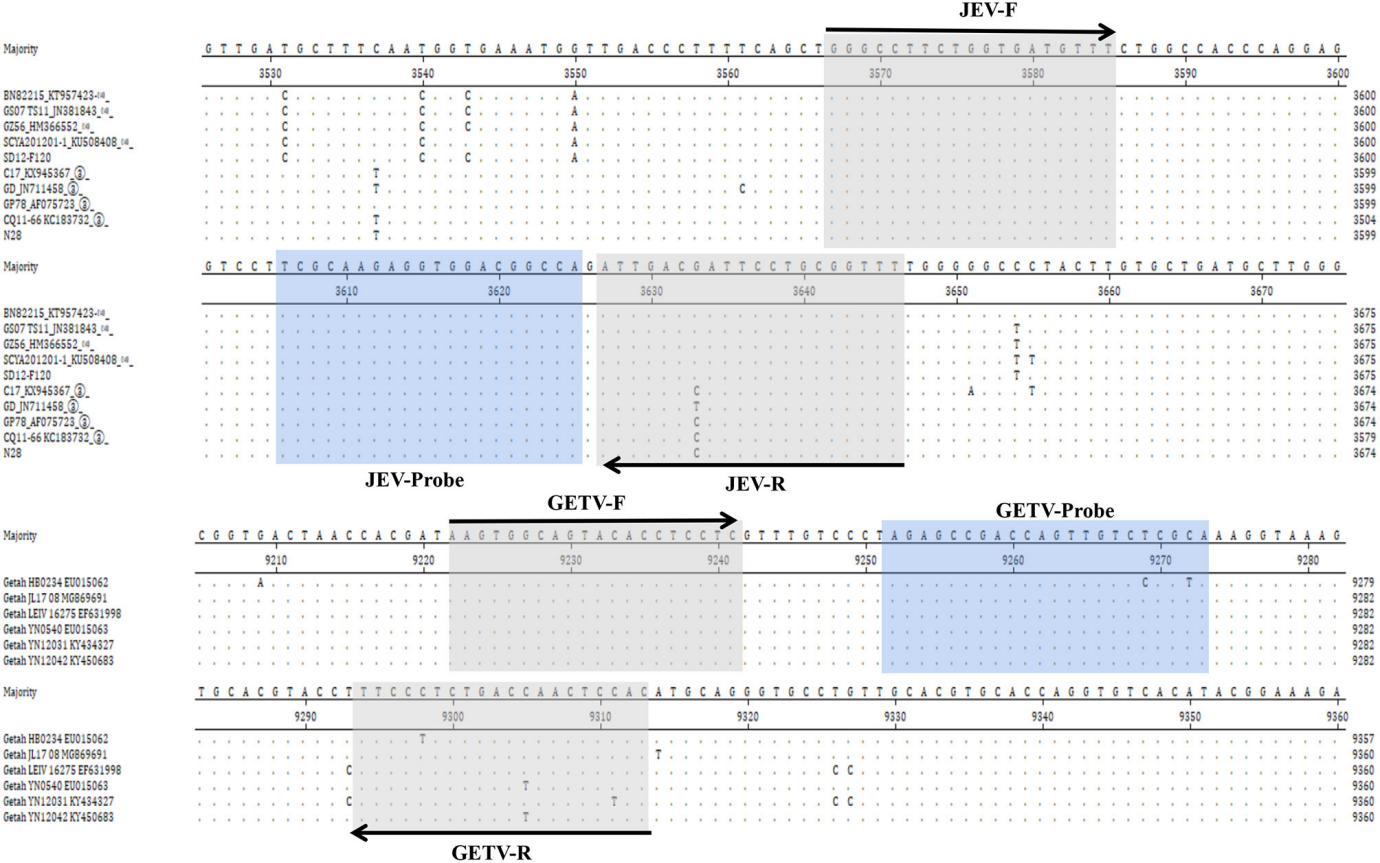
Comparison of the *NS1* gene of different strains of JEV and the *E2* gene of different strains of GETV and the positions of primers and TaqMan probes in the viral genomes. GenBank accession numbers are shown in parentheses. Dots (·) indicate identical bases.

After comparing the whole genome sequences of JEV and GETV, respectively, we decided to design primers and probes in the conserved regions of the *NS1* gene of JEV and the *E2* gene of GETV. We designed three sets of JEV primers and probes and two sets for GETV. Before the experiment, we screened the primers and probes, optimized their melting temperatures and concentrations, and finally selected two sets of primers and probes as the best combination. The detailed sequences and specific locations are shown in Table 1 and Figure 1. Using Primer-Blast (NCBI) to analyze the primers and probes revealed high conservation and high specificity of the two sets of primers and probes, meaning that they could be used for the specific detection of JEV and GETV. A single FAM or HEX fluorescent signal, respectively, could be detected by the duplex TaqMan RT-qPCR for N28 (JEV) and M1 (GETV).

### Standard Curves for JEV and GETV

**Figure 2:**
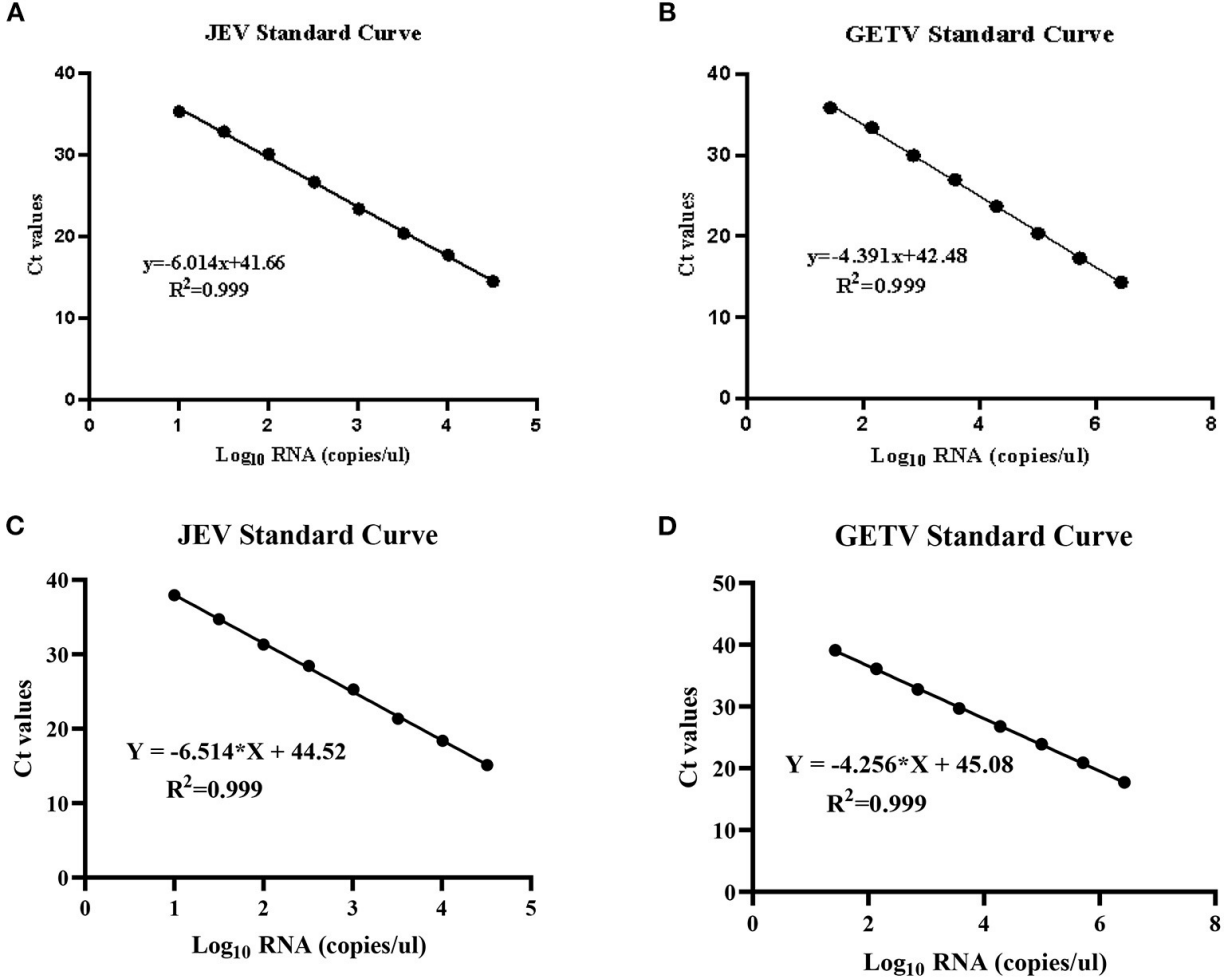
Standard curves for duplex TaqMan RT-qPCR. **(A)** Standard curve for JEV in two-step RT-qPCR. **(B)** Standard curve for GETV in two-step RT-qPCR. **(C)** Standard curve for JEV in one-step RT-qPCR. **(D)** Standard curve for GETV in one-step RT-qPCR.

We separately constructed JEV and GETV strain plasmids using pMD19-T vector to establish standard curves. The plasmids were named JEV-NS1-T and GETV-E2-T. In order to achieve a reliable calibration curve, JEV-NS1-T and GETV-E2-T were used in the linear range of 10^9^ ~ 10^1^ copies/μL RNA copy (10-fold) in real-time multiplex RT-PCR assays (two-step and one-step RT-qPCR). In two-step RT-qPCR, the linear regression equation of the JEV standard curve was y=-6.014x+41.66 (R^2^ = 0.999) (Figure 2A); the linear regression equation of the GETV standard curve was y = −4.391x+38.53 (R^2^ = 0.999) (Figure 2B). And in one-step RT-qPCR, the linear regression equations for the standard curves of JEV and GETV were y = −6.514x+44.52 (R^2^ = 0.999) (Figure 2C) and y = −4.686x+44.12 (R^2^ = 0.999) (Figure 2D). The correlation coefficients (R^2^) of the two viruses in the two-step and the one-step method were both 0.999, indicating that the two methods have high reliability for detecting the two viruses. In the subsequent multiplex real-time RT-PCR tests, we used the standard curves to quantify the number of genomic RNA copies of JEV and GETV, respectively, in clinical samples.

### Specificity of One-Step TaqMan Real-Time RT-PCR

**Figure 3:**
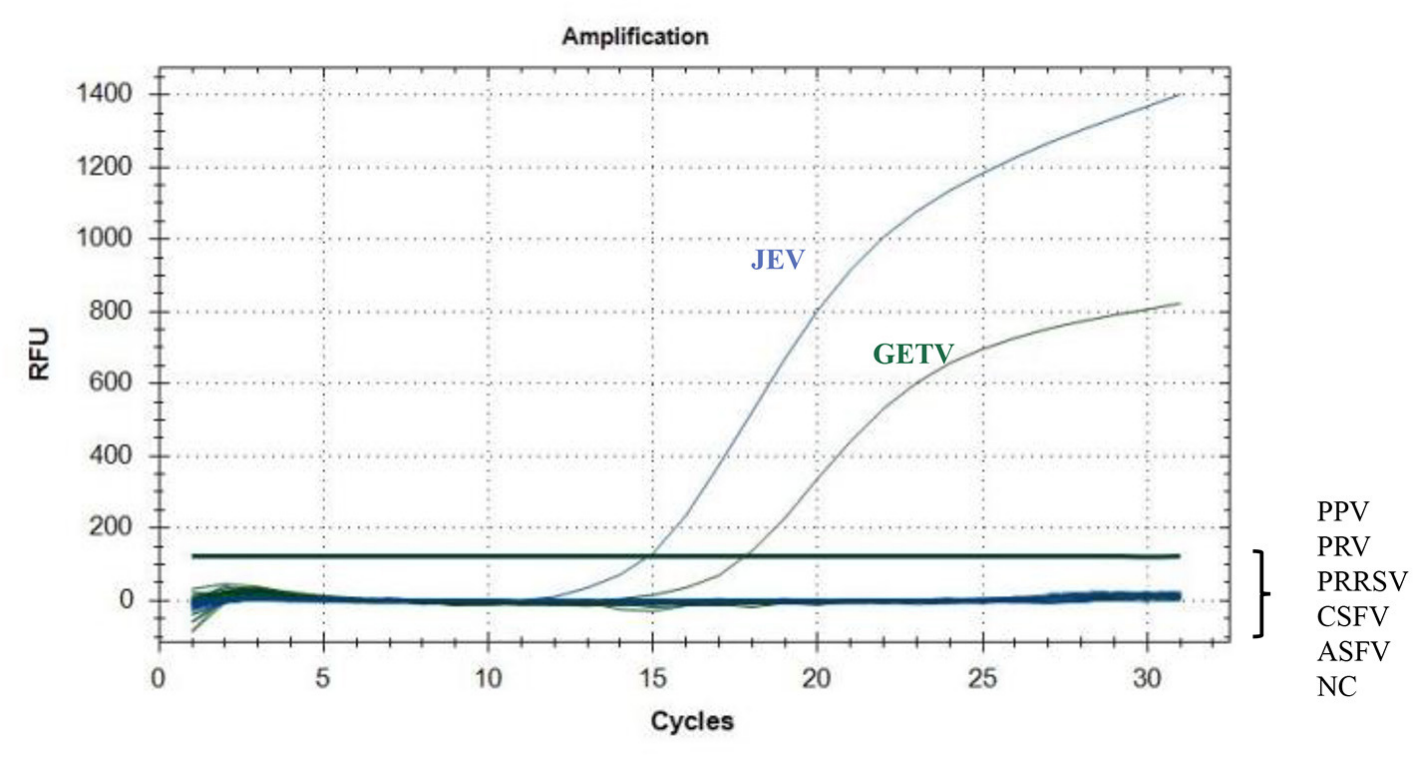
Specific amplification curves for JEV and GETV. Two specific fluorescent curves were observed in the duplex TaqMan RT-qPCR assay using RNA mixtures, representing JEV and GETV. FAM/HEX fluorescent signals specific for JEV and GETV, were collected only when the JEV and GETV isolates were used as templates. No FAM/HEX signal was observed when the samples contained other viruses.

In addition to JEV and GETV, CSFV, PPV, PRV, PRRSV, and ASFV are also prevalent in pigs. Therefore, RNA of CSFV and PRRSV and the genomes of PPV, PRV and ASFV were used to determine the specificity of the assay (Figure 3). When the template was JEV, only FAM signals were detected; when the template was GETV, HEX signals were detected but FAM signals could not be detected. This showed that the method had the ability to effectively distinguish between the two viruses, JEV and GETV. When other viruses were used as templates, no positive signals for FAM and HEX could be detected, indicating that this method had high specificity for both JEV and GETV.

### Sensitivity of the Duplex TaqMan RT-qPCR Assay

**Figure 4:**
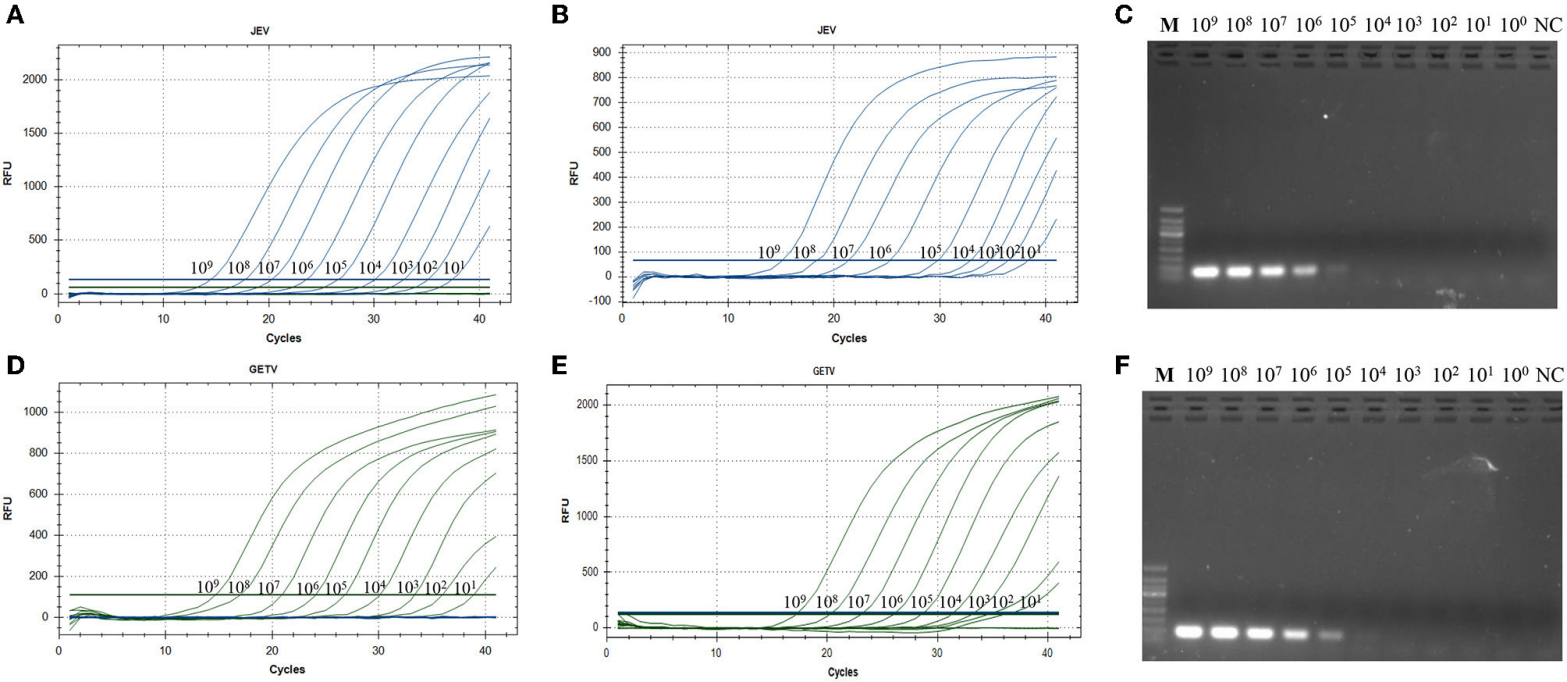
**(A)** Amplification curve showing the sensitivity of the duplex TaqMan-based real-time PCR to detect JEV. **(B)** Sensitivity of the singleplex TaqMan-based real-time PCR to detect JEV. **(C)** Sensitivity of conventional PCR for JEV. **(D)** Amplification curve showing the sensitivity of the duplex TaqMan-based real-time PCR to detect GETV. **(E)** Sensitivity of the singleplex TaqMan-based real-time PCR to detect GETV. **(F)** Sensitivity of conventional PCR for GETV. All RNA standards were serially diluted 10 times (10^9^ copies/μL~10^0^ copies/μL).

Using 10-fold serial dilutions of JEV and GETV standards as templates, we used the double TaqMan RT-qPCR method, singleplex TaqMan-based RT-qPCR and the traditional RT-PCR method for detection of JEV and GETV to compare the differences in sensitivity of the three methods. The traditional RT-PCR method has the lowest sensitivity. The One-step duplex TaqMan RT-qPCR assay and singleplex TaqMan RT-qPCR medthod were found to be highly sensitive with detection limits of 1,000 genomic copy for both JEV and GETV strains, and its sensitivity was 100 times and 10 time higher than traditional RT-PCR assays, respectively (Table 2, Figure 4).

**Table 2:** Comparison of the sensitivity of duplex TaqMan RT-qPCR, singleplex TaqMan RT-qPCR and conventional RT-PCR.

**Viruses**	**Copy number**	**Dupelx RT-qPCR**		**Singleplex RT-qPCR**		**RT-PCR**
		**Ct value**		**Ct value**		
JEV	10^9^	14.47615390	+	15.10934790	+	+
	10^8^	17.71620136	+	18.38510417	+	+
	10^7^	20.35256067	+	21.33723624	+	+
	10^6^	23.33982386	+	25.27014159	+	+
	10^5^	26.64371346	+	29.81450521	+	+
	10^4^	30.06685677	+	32.91795920	+	−
	10^3^	32.81907573	+	34.69597800	+	−
	10^2^	35.26345947	−	37.94815368	−	−
	10^1^	37.25804602	−	40.03301929	−	−
	10^0^	NaN	−	NaN	−	−
GETV	10^9^	14.33174411	+	17.71367265	+	+
	10^8^	17.28760469	+	20.88617182	+	+
	10^7^	20.33785731	+	23.89212114	+	+
	10^6^	23.70719733	+	26.77344945	+	+
	10^5^	26.95672766	+	29.69046728	+	+
	10^4^	29.96147202	+	32.05405170	+	+
	10^3^	33.43240883	+	34.08649457	+	−
	10^2^	35.84064952	−	36.46553223	−	−
	10^1^	37.49041068	−	38.74502891	−	−
	10^0^	NaN	−	NaN	−	−

*+, positive; −, negative.*

*FAM channel Ct value ≤ 35 and typical amplification curve indicates JEV positive; HEX channel Ct value ≤ 35 and typical amplification curve indicates GETV positive*.

### Intra-Assay and Inter-Assay Reproducibility of the Duplex TaqMan RT-qPCR Assay

Variations in the PCR amplicon Ct in the assays are shown in Table 3. The intra-assay and inter-assay coefficients of variation for JEV and GETV were both lower than 1.6% and 3.1%. This proved that the method had good reproducibility for the detection of specific viruses.

**Table 3:** Reproducibility of the duplex TaqMan RT-qPCR assay evaluated using ssRNA standards of JEV and GETV.

**Virus standards**	**Copy number**	**Intra-assay**		**Inter-assay**	
		**CT value (mean±SD)**	**CV(%)**	**CT value (mean±SD)**	**CV(%)**
JEV	10^9^	14.5036 ± 0.0837	0.2018	14.4723 ± 0.1385	0.9571
	10^8^	17.8399 ± 0.1237	0.6038	17.3602 ± 0.0363	0.2095
	10^7^	20.3033 ± 0.0492	0.2184	19.3400 ± 0.2234	1.1553
	10^6^	23.4529 ± 0.1131	0.4640	22.1684 ± 0.0794	0.3582
	10^5^	26.6105 ± 0.0510	0.1678	25.6455 ± 0.1215	0.4742
	10^4^	30.1184 ± 0.0522	0.1722	29.6568 ± 0.4610	1.3742
	10^3^	33.2563 ± 0.4373	1.1386	33.5397 ± 0.4677	1.5024
	10^2^	34.9788 ± 0.2846	1.0708	36.4413 ± 0.6512	1.7872
	10^1^	36.6428 ± 0.6152	1.5160	38.7541 ± 0.6371	1.6440
	10^0^	NaN	NaN	NaN	NaN
	NC	NaN	NaN	NaN	NaN
GETV	10^9^	14.2903 ± 0.1187	1.0069	13.8113 ± 0.2437	1.6085
	10^8^	17.2017 ± 0.1852	0.9328	15.6568 ± 0.5387	2.9806
	10^7^	20.3197 ± 0.0522	0.0965	17.6133 ± 0.6198	3.0796
	10^6^	23.7612 ± 0.0769	0.2879	21.4036 ± 0.3020	1.2729
	10^5^	26.9223 ± 0.0637	0.2053	24.1969 ± 0.7694	2.7903
	10^4^	30.2232 ± 0.2618	0.7958	29.0044 ± 0.3331	2.2680
	10^3^	33.8922 ± 0.4197	1.3016	32.2797 ± 0.5314	1.4728
	10^2^	36.2325 ± 0.4921	1.2432	33.7941 ± 0.4300	1.2831
	10^1^	37.0263 ± 0.5359	1.3029	38.3502 ± 1.6997	2.5425
	10^0^	NaN	NaN	NaN	NaN
	NC	NaN	NaN	NaN	NaN

### Comparison of Duplex TaqMan RT-qPCR With RT-PCR and Virus Isolation

We tested 18 mosquito pools obtained from 1834 mosquitoes collected from the field using duplex TaqMan RT-qPCR and conventional RT-PCR assays. Four mosquito pools tested positive for JEV using the dual RT-qPCR method, while two mosquito pools tested positive for GETV. Traditional PCR only detected positive JEV in three mosquito pools and positive GETV in two mosquito pools. Among all mosquito pools, JEV could be separated from one mosquito pools, while GETV could be separated in only one mosquito pool (Table 4).

**Table 4:** Comparison of the performance of RT-qPCR, RT-PCR, and virus isolation for simultaneous detection of JEV and GETV in clinical and experimental samples.

**Species**	**Location**	**Year**	**Sample**	**RT-PCR**	**Virus isolation**	**Specific detect system (Ct/Virus)**
Mosquitoes	Shanghai	2016	20160612	−	−	−
			20160623	−	−	−
			20160624	+	+	FAM 13.54/JEV
			20160627	−	−	−
			20160628	−	−	FAM 32.85/JEV
			20160630	+	+	HEX 21.58/GETV
			20160712	−	−	−
			20160714	−	−	−
			20160715	−	−	−
			20160727	+	−	FAM 30.33/JEV
			20160809	−	−	−
			20160811	−	−	−
			20160813	+	−	FAM 27.68/JEV
			20160814	−	−	−
			20160816	−	−	−
			20160819	−	−	−
			20160820	+	−	HEX 29.59/GETV
			20160825	−	−	−
Pig	Shanghai	2016	Blood	−	−	−
			Blood	−	−	−
			Blood	+	+	HEX 25.33/GETV
			Blood	+	−	FAM 28.85/JEV
			Blood	−	−	−
			Brain	−	−	FAM 33.75/JEV
			Brain	−	−	−
			Brain	−	−	−
			Brain	+	+	FAM 16.75/JEV
			Brain	−	−	−
			Brain	−	−	HEX 34.23/GETV
			Liver	−	−	−
			Spleen	+	−	FAM 30.75/JEV
			Kidney	−	−	−
			Spleen	−	−	−

*+, positive; −, negative.*

*# Ct value of FAM channel ≤ 35 with a typical amplification curve and Ct value of HEX channel > 35 or none without a typical amplification curve indicates samples are JEV positive; otherwise samples are GETV positive*.

Among the 15 pig tissue and blood samples tested using the duplex TaqMan RT-qPCR assay, two samples were detected as GETV positive (Ct ≤ 35) and four samples were detected as JEV positive (Ct ≤ 35). Three samples and one sample tested positive for JEV and GETV, respectively, by traditional RT-PCR methods; while virus isolation could only detect one GETV and one JEV positive samples (Table 4).

Overall, our results indicated that duplex TaqMan RT-qPCR was more sensitive than conventional RT-PCR or virus isolation. All of the JEV-positive samples were confirmed by DNA sequencing, and the sequencing results were consistent with the one-step duplex TaqMan RT-qPCR results.

### Application and Detection of Clinical Samples

A total of 331 clinical samples from Shanghai, Xinjiang and Yunnan, China, including 115 pig samples (77 blood samples and 38 brains) and 21,647 female mosquitoes in 216 mosquito pools. Duplex TaqMan RT-qPCR was used to detect and identify JEV and GETV (Table 5), the duplex TaqMan RT-qPCR test showed that 9.6% (32/331) of the samples were JEV, 1.5% (5/331) were GETV, 88.8% (290/331) were negative. These results indicate that both JEV and GETV are prevalent in pigs or mosquitoes in China.

**Table 5:** Detection of JEV and GETV in clinical samples by duplex TaqMan RT-qPCR in mosquitos and pigs during 2017–2018.

**Location**	**Type of sample**	**NO. of samples**	**NO. of samples positive**	
			**JEV**	**GETV**
Xinjiang	Mosquito pools	73	3	0
	Blood	38	0	0
	Brain	12	0	0
Yunnan	Mosquito pools	64	14	2
	Blood	21	3	1
	Brain	17	2	0
Shanghai	Mosquito pools	79	7	1
	Blood	18	3	0
	Brain	9	0	1
Total		331	32	5

## Discussion

JEV is a serious mosquito-borne pathogen that can infect a variety of animals. JEV has long been one of the major public health problems in Asian countries including China (22, 23). However, in recent years, with the continuous expansion of the geographic scope of GETV in mainland China, GETV has become a “new arbovirus” that has invaded mainland China (6). GETV and JEV are often present as mixed infections in pigs and mosquitoes because the vectors of both viruses are *Culex* spp. mosquitoes and the epidemic areas also overlap greatly (10, 24). Furthermore, the similar clinical symptoms of JEV and GETV have brought new difficulties for early detection and prevention. Pigs and mosquitoes are considered to be the most important natural amplification hosts of JEV and GETV, and they play a primary role in the spread of the virus (17). Thus, developing rapid differentiation assays for JEV and GETV from field samples has become imperative for virological surveillance, decision making, and developing/evaluating JEV and GETV control strategies. In this study, we developed a specific and sensitive duplex TaqMan RT-qPCR method for the rapid detection and differentiation of JEV and GETV from pig and mosquito samples.

The traditional gold standard for detecting JEV and GETV is to use virus isolation and identification, but this method is time-consuming, laborious, and expensive when testing a large number of clinical samples. Serological tests such as virus neutralization tests and ELISA are also routine methods for diagnosing JEV and GETV (18, 25). However, serological testing also has some shortcomings, including that the results of antibody testing may be affected by viral cross-reactions, and this method is not sensitive to early detection of viral infections (26).

Nucleic acid detection methods have become commonly used in experiments and clinical diagnoses due to their advantages in sensitivity and specificity over traditional detection techniques. Among them, quantitative real-time PCR technology not only maintains the advantages high sensitivity obtained with traditional PCR technology, but also overcomes the shortcomings of false positives and inaccurate quantification, and has good experimental reproducibility, accuracy, and specificity (27).

An RT-qPCR based on SYBR Green has been established for the detection of JEV and GETV, and SYBR Green has been widely used to detect and quantify JEV and GETV in clinical samples (20, 28). However, due to its ability to bind any double-stranded DNA, nonspecific binding can lead to low specificity of results (29). In contrast, the RT-qPCR based TaqMan method offers stronger specificity and higher sensitivity (30). Previously, researchers established a RT-qPCR based TaqMan detection method to detect JEV and GETV in pig and mosquito samples (15, 30). However, few studies have used TaqMan chemical methods to identify and quantitatively detect JEV and GETV in field specimens. Many studies have established TaqMan-based RT-qPCR methods for simultaneous identification and detection of multiple viruses, including the detection of WNV (West Nile virus) and JEV; porcine enteric coronaviruses; and different genotypes of JEV (17, 21, 31). It is therefore feasible to establish a Taqman-based RT-qPCR method for simultaneous identification and detection of JEV and GETV.

In this study, we designed two pairs of TaqMan BHQ probes and primers against the GETV *E2* gene and the JEV *NS1* gene and developed a TaqMan probe-based dual RT-qPCR method that can distinguish and detect JEV and GETV. The new method had good specificity, no cross-reactivity to other common viruses (including CSFV, PPV, PRV, PRRSV, and ASFV), and could differentiate JEV from GETV well; it was also more sensitive than traditional PCR. In addition, the RT-qPCR based on dual TaqMan probes showed good repeatability in intra-assay and inter-assay repeatability tests.

We established a one-step duplex TaqMan RT-qPCR assay, which can quickly and accurately identify and detect JEV and GETV. This assay be used for on-site monitoring and identification of JEV and GETV in captured mosquito and pig samples. It therefore provides valuable tools for the rapid detection, clinical diagnosis, and epidemiological investigation of these two viruses.

## Data Availability Statement

The original contributions presented in the study are included in the article/**Supplementary Material**, further inquiries can be directed to the corresponding authors.

## Ethics Statement

The animal study was reviewed and approved by Committee of Shanghai Veterinary Research Institute, Chinese Academy of Agricultural Sciences, Shanghai.

## Author Contributions

YZ, QS, BL, YQ, JZ, and KL: validation. YL, YZ, YY, and JW: formal analysis. YY, YZ, JZ, and JW: investigation. BL, ZG, and YQ: writing—review and editing. DS, ZG, and JW: methodology. ZM, JW, and PL: conceptualization. ZM: project administration. JW and ZM: funding acquisition. PL and JW: writing—original draft. All authors contributed to the article and approved the submitted version.

## Funding

This study was supported by Shanghai Agriculture Applied Technology Development Program, China (no. X2021-02-08-00-12-F00770 awarded to JW), the Shanghai Science and Technology Commission (no. 22N41900400 awarded to ZM), the Natural Science Foundation of Shanghai (no. 19ZR1469000 awarded to JW), and the Central Public-interest Scientific Institution Basal Research Fund (no. Y2020PT40 awarded to JW).

## Conflict of Interest

The authors declare that the research was conducted in the absence of any commercial or financial relationships that could be construed as a potential conflict of interest.

## Publisher's Note

All claims expressed in this article are solely those of the authors and do not necessarily represent those of their affiliated organizations, or those of the publisher, the editors and the reviewers. Any product that may be evaluated in this article, or claim that may be made by its manufacturer, is not guaranteed or endorsed by the publisher.

## Supplementary Material

Supplementary Figure 1 Result of JEV/GETV probe concentration optimization to TaqMan real-time PCR. **(A)** Amplification curves of JEV at different probe concentrations. **(B)** Amplification curves of GETV at different probe concentrations.

## References

[1] Shi N Liu H Li Lx Hu B Zhang L Zhao Cf. Development of a TaqMan probe-based quantitative reverse transcription PCR assay for detection of Getah virus RNA. Arch Virol. (2018) 163:2877–81. 10.1007/s00705-018-3927-2 29987379

[2] Li Y-Y Liu H Fu S-H Li X-L Guo X-F Li M-H. From discovery to spread: the evolution and phylogeny of Getah virus. Infect Genet Evol. (2017) 55:48–55. 10.1016/j.meegid.2017.08.016 28827175

[3] Yang T Li R Hu Y Yang L Zhao D Du L. An outbreak of Getah virus infection among pigs in China, 2017. Transbound Emerg Dis. (2018) 65:632–7. 10.1111/tbed.12867 29575687

[4] Scherer WF Funkenbusch M Buescher EL and Izumit. Sagiyama virus, a new group A arthropod-borne virus from Japan. I. Isolation, immunologic classification, ecologic observations. Am. J. Trop. Med. (1962) 11:255–68. 10.4269/ajtmh.1962.11.255 14498263

[5] Nemoto M Bannai H Tsujimura K Kobayashi M Kikuchi T Yamanaka T. Getah virus infection among racehorses, Japan, 2014. Emerg Infect Dis. (2015) 21:883–5. 10.3201/eid2105.141975 25898181 PMC4412242

[6] Liu H Zhang X Li L Shi N Sun X Liu Q. First isolation and characterization of Getah virus from cattle in northeastern China. BMC Vet Res. (2019) 15:320. 10.1186/s12917-019-2061-z 31488162 PMC6729113

[7] Wang A Zhou F Chang H Wang X Chen L Cui D. Molecular detection, isolation and identification of the porcine Getah virus from pig herds in four provinces, China. Chin J Microbiol Immunol. (2018) 34:522–32. 10.3389/fvets.2020.552517 33344520 PMC7744783

[8] Kulkarni R Sapkal GN Kaushal H Mourya DT. Japanese encephalitis: a brief review on indian perspectives. Open Virol J. (2018) 12:121–30. 10.2174/1874357901812010121 30288200 PMC6142657

[9] Campbell GL Hills SL Fischer M Jacobson JA Hoke CH Hombach JM. Estimated global incidence of Japanese encephalitis: a systematic review. Bull World Health Organ. (2011) 89:766–74. 10.2471/BLT.10.085233 22084515 PMC3209971

[10] Vaughn DW Hoke CHJr. The epidemiology of Japanese encephalitis: prospects for prevention. Epidemiol Rev. (1992) 14:197–221. 10.1093/oxfordjournals.epirev.a036087 1337744

[11] Dhanze H Kumar MS Singh V Gupta M Bhilegaonkar KN Kumar A. Detection of recent infection of Japanese encephalitis virus in swine population using IgM ELISA: a suitable sentinel to predict infection in humans. J Immunol Methods. (2020) 486:112848. 10.1016/j.jim.2020.112848 32891615

[12] Scherer WF Moyer JT Izumi T Gresser I McCown J. Ecologic studies of Japanese encephalitis virus in Japan. VI Swine infection. Am J Trop Med. (1959) 8:698–706. 10.4269/ajtmh.1959.8.698 14442656

[13] Chai C Wang Q Cao S Zhao Q Wen Y Huang X. Serological and molecular epidemiology of Japanese encephalitis virus infections in swine herds in China, 2006-2012. J Vet Sci. (2018) 19:151–5. 10.4142/jvs.2018.19.1.151 28693301 PMC5799393

[14] Tajima S Kotaki A Yagasaki K Taniwaki T Moi ML Nakayama E. Identification and amplification of Japanese encephalitis virus and Getah virus propagated from a single porcine serum sample: a case of coinfection. Arch Virol. (2014) 159:2969–75. 10.1007/s00705-014-2152-x 24986716

[15] Bharucha T Sengvilaipaseuth O Vongsouvath M Vongsouvath M Davong V Panyanouvong P. Development of an improved RT-qPCR Assay for detection of Japanese encephalitis virus (JEV) RNA including a systematic review and comprehensive comparison with published methods. PLoS ONE. (2018) 13. 10.1371/journal.pone.0194412 PMC5865736 29570739

[16] Wei JC Wang X Zhang JJ Guo S Pang LL Shi K. Partial cross-protection between Japanese encephalitis virus genotype I and III in mice. PLOS Negl Trop Dis. (2019) 13. 10.1371/journal.pntd.0007601 PMC6693775 31374086

[17] Wang X Guo S Hameed M Zhang J Pang L Li B. Rapid differential detection of genotype I and III Japanese encephalitis virus from clinical samples by a novel duplex TaqMan probe-based RT-qPCR assay. J Virol Methods. (2020) 279. 10.1016/j.jviromet.2020.113841 32105753

[18] Moore CE Blacksell SD Taojaikong T Jarman RG Gibbons RV Lee SJ. A prospective assessment of the accuracy of commercial IgM ELISAs in diagnosis of Japanese encephalitis virus infections in patients with suspected central nervous system infections in Laos. Am J Trop Med Hyg. (2012) 87:171–8. 10.4269/ajtmh.2012.11-0729 22764310 PMC3391045

[19] Wu T Fu S Yin Q Zhao J Li F He Y. Establishment of TaqMan RT-qPCR assay for the detection Getah virus. J Clin Virol. (2021) 35:205–8. 10.1038/s41598-021-99734-7 34625631 PMC8501081

[20] Xia Y- h Shi Z-,c Wang X-,w Li Y-,t Wang Z Chang H- t. (2021). Development and application of SYBR Green I real-time quantitative reverse transcription PCR assay for detection of swine Getah virus. Mol Cell Probes. 57:101730. 10.1016/j.mcp.2021.101730 33848593

[21] Huang X Chen J Yao G Guo Q Wang J Liu G. A TaqMan-probe-based multiplex real-time RT-qPCR for simultaneous detection of porcine enteric coronaviruses. Appl Microbiol Biotechnol. (2019) 103:4943–52. 10.1007/s00253-019-09835-7 31025076 PMC7080015

[22] Zhang JS Zhao QM Guo XF Zuo SQ Cheng JX Jia N. (2011). Isolation and genetic characteristics of human genotype 1 Japanese Encephalitis Virus, China, 2009. Plos ONE. 6(1):e16418. 10.1371/journal.pone.0016418 PMC3026811 21283590

[23] Gould EA Coutard B Malet H Morin B Jamal S Weaver S. Understanding the alphaviruses: Recent research on important emerging pathogens and progress towards their control. Antivir Res. (2010) 87:111–24. 10.1016/j.antiviral.2009.07.007 19616028 PMC7114216

[24] Lu G Chen R Shao R Dong N Liu W Li S. Getah virus: an increasing threat in China. J Infect. (2020) 80:356–8. 10.1016/j.jinf.2019.11.016 31790706

[25] Meng J He Y Yang S Li N Zeng S Xu T. Establishment and application of an indirect ELISA for detection of swine Getah disease. Chin J Vet. (2020) 50:962–8. 10.1111/tbed.14267 34328270

[26] Hirota J Nishi H Matsuda H Tsunemitsu H Shimizu S. Cross-reactivity of Japanese encephalitis virus-vaccinated horse sera in serodiagnosis of West Nile virus. J Vet Med Sci. (2010) 72:369–72. 10.1292/jvms.09-0311 19996564

[27] Alvarez-Diaz DA Quintero PA Pelaez-Carvajal D Ajami NJ Usme-Ciro JA. Novel pan-serotype control RNA for dengue virus typing through real-time reverse transcription-polymerase chain reaction. J Virol Methods. (2019) 271:113677. 10.1016/j.jviromet.2019.113677 31195032

[28] Zhu Z Zhang X Teng Z Zhao B Shao J Li Y. Development and primary application of a SYBR Green I real-time polymerase chain reaction for the detection of Japanese encephalitis virus. Zhongguo yu fang yi xue hui xi lie za zhi. (2007) 18:215–8. 10.1016/j.jviromet.2007.02.011 17403544

[29] Kedrak-Jablonska A Budniak S Krupa M Szczawinska A Reksa M Szulowski K. Detection of Listeria spp. and Listeria monocytogenes in biological samples by SYBR Green I and TaqMan probe-based real-time PCRs. J Vet Sci. (2017) 61:427–32. 10.1515/jvetres-2017-0069 29978105 PMC5937340

[30] Sam S-S Teoh B-T Chee C-M Mohamed-Romai-Noor N-A Abd-Jamil J Loong S-K. A quantitative reverse transcription-polymerase chain reaction for detection of Getah virus. Scient Rep. (2018) 8:17632. 10.1038/s41598-018-36043-6 30518924 PMC6281642

[31] Shirato K Miyoshi H Kariwa H Takashima I. Detection of West Nile virus and Japanese encephalitis virus using real-time PCR with a probe common to both viruses. J Virol Methods. (2005) 126:119–25. 10.1016/j.jviromet.2005.02.001 15847927

