# Supplementary material for: Rapid Differential Detection of Japanese Encephalitis Virus and Getah Virus in Pigs or Mosquitos by a Duplex TaqMan Real-Time RT-PCR Assay
Source: Front Vet Sci. 2022 Apr 7;9:839443. doi: 10.3389/fvets.2022.839443 (PMC9023051; doi:10.3389/fvets.2022.839443)
Supplement: Supplementary Figure 1 — Result of JEV/GETV probe concentration optimization to TaqMan real-time PCR. (A) Amplification curves of JEV at different probe concentrations. (B) Amplification curves of GETV at different probe concentrations. [file Presentation_1.pptx]

## Slide 1
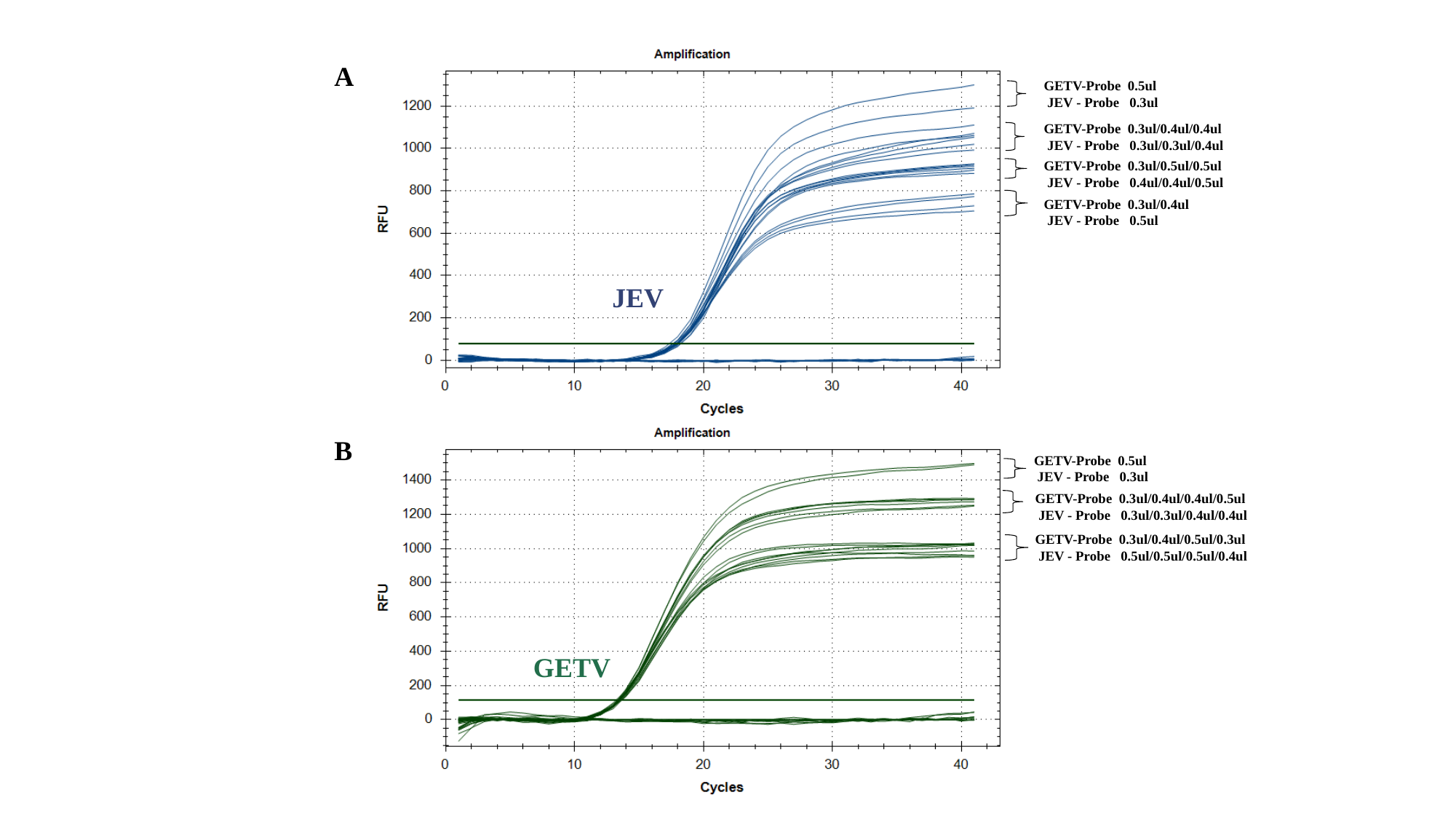

A
GETV-Probe 0.5ul
 JEV - Probe 0.3ul
GETV-Probe 0.3ul/0.4ul/0.4ul
 JEV - Probe 0.3ul/0.3ul/0.4ul
GETV-Probe 0.3ul/0.5ul/0.5ul
 JEV - Probe 0.4ul/0.4ul/0.5ul
GETV-Probe 0.3ul/0.4ul
 JEV - Probe 0.5ul
JEV
B
GETV-Probe 0.5ul
 JEV - Probe 0.3ul
GETV-Probe 0.3ul/0.4ul/0.4ul/0.5ul
 JEV - Probe 0.3ul/0.3ul/0.4ul/0.4ul
GETV-Probe 0.3ul/0.4ul/0.5ul/0.3ul
 JEV - Probe 0.5ul/0.5ul/0.5ul/0.4ul
GETV
